# Genome-wide analysis of extended-spectrum beta-lactamase-producing *Escherichia coli* from seafood in Bangladesh: population structure, resistome, virulome, and global dissemination patterns

**DOI:** 10.3389/fmicb.2026.1737712

**Published:** 2026-02-06

**Authors:** Naeem Ahammed Ibrahim Fahim, Afsan Sarwer, Zannatul Firdous, Md. Liton Rana, Md. Saiful Islam, Saifur Rahman, Amrita Pondit, Jayedul Hassan, Timothy R. Walsh, Refath Farzana, Md. Tanvir Rahman

**Affiliations:** 1 Department of Microbiology and Hygiene, Faculty of Veterinary Science, Bangladesh Agricultural University, Mymensingh, Bangladesh; 2 National Engineering Research Center of Industrial Wastewater Detoxication and Resource Recovery, Research Center for Eco-Environmental Sciences, Chinese Academy of Sciences, Beijing, China; 3 University of Chinese Academy of Sciences, Beijing, China; 4 Department of Animal Science, University of California, Davis, Davis, CA, United States; 5 Department of Biology, Ineos Oxford Institute for Antimicrobial Resistance, University of Oxford, Oxford, United Kingdom

**Keywords:** beta-lactamase, *Escherichia coli*, global-phylogeny, seafood, whole-genome-sequencing, antimicrobial resistance, one health, virulence

## Abstract

**Objectives:**

This study aimed to identify multilocus sequence type (MLST), serotype, average nucleotide identity (ANI), antimicrobial resistance genes (ARGs), virulence genes (VGs), and mobile genetic elements (MGEs) from whole-genome sequences of 10 *Escherichia coli* isolated from seafood in Bangladesh and compared them with the global datasets of beta-lactamase-producing *E. coli*.

**Methods:**

Ten *E. coli* isolates (crab = 3, shrimp = 1, tuna = 6) were subjected to whole-genome sequencing using Oxford Nanopore Technologies (Oxford, UK). In-silico bioinformatics analyses were performed using online tools and Linux-based commands. A systematic advanced search in PubMed identified 722 global genomes of beta-lactamase-producing *E. coli* for comparative analysis. A population structure and global phylogeny were constructed to illustrate the current status of beta-lactamase-producing *E. coli* from diverse sources (seafood, human, aquatic, wastewater, and environmental) across countries, based on their STs, ARGs, VGs, MGEs, and serotypes.

**Results:**

Bioinformatics analysis revealed that most isolates displayed unique sequence types (STs) and core genome sequence types (cgSTs), while three isolates shared both ST1431 and cgST104784, indicating close genetic relatedness supported by ANI analysis. In shrimp and tuna isolates, both O and H antigens were detected, whereas crab isolates carried either O or H antigens. Pangenome analysis identified 56.4% strain-specific genes, 34.2% dispensable genes, and 9.4% core genes, with functions categorized into clusters of orthologous groups (COGs). Several ARGs, including beta-lactamase genes (*CTX-M-15, AmpC, bla*_DHA-1_), were detected across isolates, with crabs harboring the highest number. The VGs were more common in tuna isolates. Plasmids were only detected in crabs (Col440I, IncFIA, IncFIB(pHCM2), and ColRNAI) carrying *qnrB4*, *dfrA17*, *qacE*, *mph(A)*, *sul1*, *bla*_DHA-1_ ARGs, but were absent in shrimp and tuna. Population structure analysis showed that ST345 in Bangladesh closely matched wastewater (Czech Republic) and human (USA) isolates. Some STs overlapped with international records, while others appeared novel, suggesting limited global distribution. Globally, wastewater and human isolates from the Czech Republic showed the greatest similarity to our strains.

**Conclusion:**

These findings underscore the potential role of seafood in disseminating beta-lactamase-producing *E. coli*, highlighting the urgent need for integrated surveillance to mitigate antimicrobial resistance risks in humans, animals, and the food chain.

## 1 Introduction

Seafood is widely consumed worldwide due to its unique sensory attributes and high nutritional value, particularly its richness in protein, minerals, and omega-3 fatty acids. Global seafood consumption has more than doubled in the past five decades, fueled by population growth, urbanization, and changes in dietary preferences (Guillen et al., 2019). However, seafood quality is influenced by multiple factors, including freshness, geographic origin, and post-harvest practices (Ejeta et al., 2024). Among foodborne commodities, seafood is particularly vulnerable to microbial contamination because of its direct contact with aquatic ecosystems that may receive untreated waste and industrial effluents. Additionally, inadequate handling, improper storage, and unhygienic processing further increase the risk of contamination of seafood (Chintagari et al., 2017). As a result, seafood can act as a reservoir and transmission vehicle for pathogenic microorganisms and antimicrobial-resistant bacteria, raising concerns for food safety, public health, and international trade (Parlapani et al., 2023).

Among seafood-borne pathogens, *Escherichia coli* is of special importance. While many strains are commensal inhabitants of the intestinal tracts of humans and animals, pathogenic variants of *E. coli* are capable of causing a wide range of illnesses, including gastroenteritis, urinary tract infections, and septicemia (Ramos et al., 2020). Its occurrence has been associated with multiple contamination routes, including the discharge of untreated human or animal waste into water bodies, the use of contaminated ice for storage, unhygienic handling during processing, and cross-contamination within distribution and retail environments (Costa, 2013). Consuming raw or undercooked seafood contaminated with *E. coli* can lead to outbreaks of foodborne illness, which may manifest as abdominal cramps, bloody or watery diarrhea, fever, nausea, and vomiting (Jnani and Ray, 2023). Previous studies have reported *E. coli* in shrimp (Immaculate et al., 2012; Loest et al., 2022; Teophilo et al., 2002), crabs (Matulkova et al., 2013) and tuna fish (Natalia et al., 2022; Sahami and Nursinar, 2019), underscoring its widespread distribution in seafood species of global commercial importance.

Beyond pathogenicity, seafood-associated *E. coli* may serve as reservoirs of antimicrobial resistance genes (ARGs) that can be horizontally transferred within the bacterial population. Horizontal gene transfer through mobile genetic elements (MGEs), such as plasmids, integrons, and transposons, facilitates the spread of resistance traits across diverse bacterial populations, thereby compounding the public health threat (Castañeda-Barba et al., 2023). Of particular concern is resistance to *β*-lactam antibiotics, largely mediated by extended-spectrum β-lactamases (ESBLs) (Bajaj et al., 2016). ESBL-producing *E. coli* compromises the efficacy of critically important antimicrobials and is frequently co-associated with resistance to non-β-lactam classes. Among the ESBL family, CTX-M-type ESBLs are globally prevalent and clinically significant worldwide. The detection of such resistance determinants in seafood is particularly alarming because it suggests the potential for transfer of resistance along the food chain, thereby bridging aquatic, animal, and human health domains.

Bangladesh is one of the world’s largest exporters of seafood (Sheikh et al., 2018), making it essential to monitor bacterial contamination and AMR and to safeguard public health and international trade. To date, no comprehensive genomic study has evaluated ESBL-producing *E. coli* in seafood in Bangladesh. Therefore, this study aimed to characterize the genomic features, antimicrobial resistance genes (ARGs), virulence genes (VGs), and mobile genetic elements (MGEs) of *E. coli* isolates obtained from shrimp, crab, and tuna, and to compare them with global datasets of ESBL-producing *E. coli* from humans, animals, and the environment. These findings will provide valuable insights into the population structure and global context of seafood-associated *E. coli* in Bangladesh.

## 2 Materials and methods

### 2.1 Ethical statement

All research methods were authorized by the Bangladesh Agricultural University (BAU) Animal Welfare and Ethics Committee in Mymensingh, Bangladesh [Approval Number: AWEEC/BAU/2023(25)]. Prior to sampling, informed permission was obtained from the vendors who sold fish in the local market.

### 2.2 Isolate selection and DNA extraction

In our previously published study (Firdous et al., 2025), we screened 102 raw seafood samples to detect extended-spectrum *β*-lactamase-producing *E. coli* (ESBL-EC), identifying ESBL-EC in 18.6% of isolates, with 69.8% exhibiting multidrug resistance and high resistance to ampicillin (100%), cefotaxime (37.2%), and ceftazidime (95.3%). The *E. coli* strains used in the present study (*n* = 8) were obtained from that first-cohort sampling, which focused on the detection of extended-spectrum β-lactamase-producing isolates resistant to ampicillin, cefotaxime, and ceftazidime, where preliminary characterization, including isolation and purification, species-level identification, and other microbiological analyses, had been completed. Additionally, two new strains (*n* = 2) were collected from a second-cohort sampling (unpublished) following the same methodological procedures and selection criteria as in our previous study. The *E. coli* isolates recovered from seafood were designated according to their source: MTR_EC02, MTR_EC03, and MTR_EC05 from crab; MTR_ES01 from shrimp; and MTR_ET01, MTR_ET06, MTR_ET08, MTR_ET09, MTR_ET11, and MTR_ET12 from tuna.

### 2.3 Whole genome sequencing and genome assembly

#### 2.3.1 Genome extraction

Genomic DNA was extracted from an overnight culture using the QIAcube (Qiagen, Hilden, Germany), and the resulting gDNA was quantified using the Qubit 3.0 (Thermo Fisher Scientific, Waltham, United States). Long-read sequencing was performed on the PromethION platform (Oxford Nanopore Technologies, Oxford, United Kingdom).

#### 2.3.2 Library prep, and sequencing

Genomic libraries were prepared using Rapid Barcoding Kit 96 V14Q20+ Kit14 (SQK-RBK114.96) according to the manufacturer’s instructions. The R10.4.1 flow cell (FLO-PRO114M) was inserted into the PromethION 2 Solo device, and barcoded DNA was loaded onto the flow cell. PromethION 2 Solo was connected to the MinKNOW Software (24.02.8) to generate the raw reads in pod5 format.

#### 2.3.3 Reads QC and genome assembly

Raw reads in pod5 format were processed utilizing the pod5 subset tool, specifically designed for partitioning pod5 files into multiple output files. Duplex basecalling was conducted with the Dorado basecaller (0.7.2), resulting in raw reads converted to fastq format, employing super accurate model, dna_r10.4.1_e8.2_400bps_sup@v4.2.0 model. Subsequently, the reads were assembled into contigs using Flye (2.9.4), followed by assembly polishing performed with Medaka (1.12.0).

#### 2.3.4 Bioinformatic analyses

The bioinformatic analyses related to genome assembly were carried out at the Advanced Research Computing (ARC) cluster, University of Oxford [arc (CPU: 48 core Cascade Lake (Intel Xeon Platinum 8,268 CPU @ 2.90GHz) Memory: 392GB) and htc (CPUs: mix of Broadwell, Haswell, Cacade Lake GPU: P100, V100, A100, RTX)].^1^ The sequencing data generated in this study have been deposited in the NCBI database under BioSample accessions SAMN52392760–SAMN52392769.

### 2.4 Population structure and global phylogeny

This study aims to provide a comprehensive analysis of ESBL-producing *E. coli* strains isolated from various sources, including human subjects, seafood, environmental samples, aquatic environments, and wastewater. To achieve this, the population structure and global phylogeny were performed to understand strain-relatedness, evolutionary history, and transmission patterns of seafood-derived *E. coli* in relation to global strains from diverse sources across various geographical locations. A systematic literature search was conducted on PubMed using the advanced search option on June 9, 2025. A total of 722 strains were selected based on different criteria, including title, full text availability, and availability of assembled genomes, using two queries, as the initial query yielded limited isolates from seafood (Supplementary Table S1E). In the first query, ((“*CTX-M-15*”[Title/Abstract] OR “*AmpC*”[Title/Abstract] OR “*blaDHA*-*1*”[Title/Abstract]) AND (“*E. coli*”[Title/Abstract] OR “*E. coli*”[Title/Abstract]) AND (“genome”[Title/Abstract] OR “WGS”[Title/Abstract] OR “genomic”[Title/Abstract] OR “whole genome sequencing”[Title/Abstract] OR “next-generation sequencing”[Title/Abstract] OR “Illumina”[Title/Abstract] OR “MinION”[Title/Abstract] OR “PacBio”[Title/Abstract]) AND (“cefotaxime”[Title/Abstract] OR “ESBL”[Title/Abstract] OR “extended-spectrum beta-lactamase”[Title/Abstract]) AND (“human”[Title/Abstract] OR “fish”[Title/Abstract] OR “fish gut”[Title/Abstract] OR “fish origin”[Title/Abstract] OR “aquatic environment”[Title/Abstract] OR “aquaculture”[Title/Abstract] OR “environment”[Title/Abstract] OR “environmental water”[Title/Abstract] OR “environmental isolates”[Title/Abstract])) (Supplementary Table S1A), and all the matadata was presented in Supplementary Table S1B.

While the second query was (“*E. coli*”[Title/Abstract] OR “*E. coli*”[Title/Abstract]) AND (“genome”[Title/Abstract] OR “WGS”[Title/Abstract] OR “genomic”[Title/Abstract] OR “whole genome sequencing”[Title/Abstract] OR “next-generation sequencing”[Title/Abstract] OR “Illumina”[Title/Abstract] OR “MinION”[Title/Abstract] OR “PacBio”[Title/Abstract]) AND (“shrimp”[Title/Abstract] OR “*Penaeus vannamei*”[Title/Abstract] OR “*Litopenaeus vannamei*”[Title/Abstract] OR “*Penaeus monodon*”[Title/Abstract] OR “crab”[Title/Abstract] OR “*Scylla serrata*”[Title/Abstract] OR “*Callinectes sapidus*”[Title/Abstract] OR “Portunus trituberculatus”[Title/Abstract] OR “tuna”[Title/Abstract] OR “*Thunnus albacares*”[Title/Abstract] OR “*Thunnus obesus*”[Title/Abstract] OR “*Thunnus thynnus*”[Title/Abstract]) (Supplementary Table S1C) and all the metadata was presented in Supplementary Table S1D. These two searches yielded a total of 30 publications from which 722 assembled genomes were downloaded from the National Center for Biotechnology Information (NCBI).

For global phylogeny, a total of 722 assembled genomes (Supplementary Table S1), one reference genome from NCBI (*E. coli* str. K-12 substr. MG1655), and selected 10 *E. coli* isolates from this study, were used to build the phylogenetic tree using Parsnp 2.0.in the Linux command, a tool designed for core genome alignment and single-nucleotide polymorphism (SNP) detection (Kille et al., 2024). However, nine genomes were excluded by the parsnp due to being much larger or shorter than the reference genome. The phylogenetic tree built based on core genome alignment of the 724 strains was then visualized using Interactive Tree of Life (iTOL v6) (Letunic and Bork, 2024). Minimal spanning trees were built on 723 genomes to show their distribution and core genomic relationships based on the housekeeping genes and 7-loci MLST profile (Supplementary Table S2) (Jolley et al., 2018)^2^, using the Grapetree tool in a Linux environment (Zhou et al., 2018).

### 2.5 Pangenome analysis

To investigate the genetic diversity among the selected *E. coli* genomes, a pangenome analysis was performed using PanExplorer (Dereeper et al., 2022). PanAcoTA, with a minimum percentage identity of 80% was employed (Perrin and Rocha, 2021). This analysis generated comparative results among the genomes and provided data and figures on core, accessory, and unique genes based on the presence or absence of genes across all genomes and their corresponding functional categories.

### 2.6 Genomic annotation and downstream analysis

Basic genomic features and elements were identified using Prokka and annotated using Bakta in the Proksee platform (Grant et al., 2023). The multilocus sequence typing (MLSTs)^3^ (Larsen et al., 2012), and cgST^4^ (Jolley and Maiden, 2010; Clausen et al., 2018) were determined using the Center for Genomic Epidemiology platform.^5^

Average nucleotide identity (ANI) values were determined by using the integrated prokaryotic genome and pan-genome analysis (IPGA) web service platform (Liu et al., 2022). The circular genomes were visualized and compared using the BLAST Ring Image Generator (BRIG) (Alikhan et al., 2011).

To detect ARGs, several platforms were used, including the Comprehensive Antibiotic Resistance Database (CARD) and CARD resistance gene identifier in proksee (Alcock et al., 2023), CARD in abricate^6^, and ResFinder (Zankari et al., 2012). The datasets of the Virulence Factors of Pathogenic Bacteria Database (VFDB) and VFDB from abricate (see footonote 6) were used to identify virulence factors (Chen et al., 2005). After each result from these analyses, the results were merged, and missing genes were added from different platforms to produce a comprehensive report. The Mobile Element Finder^8^ on the CGE and the mobileOG-db of Proksee^9^ were used to identify mobile elements, plasmids, and their associated AMR and virulence genes (VGs) (Johansson et al., 2021; Brown et al., 2022). *E. coli* serotyping was done using Ectyper^10^ (Bessonov et al., 2021) and seroTypeFinder^11^ (Joensen et al., 2015).

## 3 Result

### 3.1 Basic genomic features, MLST, cgMLST, serotype, and average nucleotide identity (ANI)

**Figure 1:**
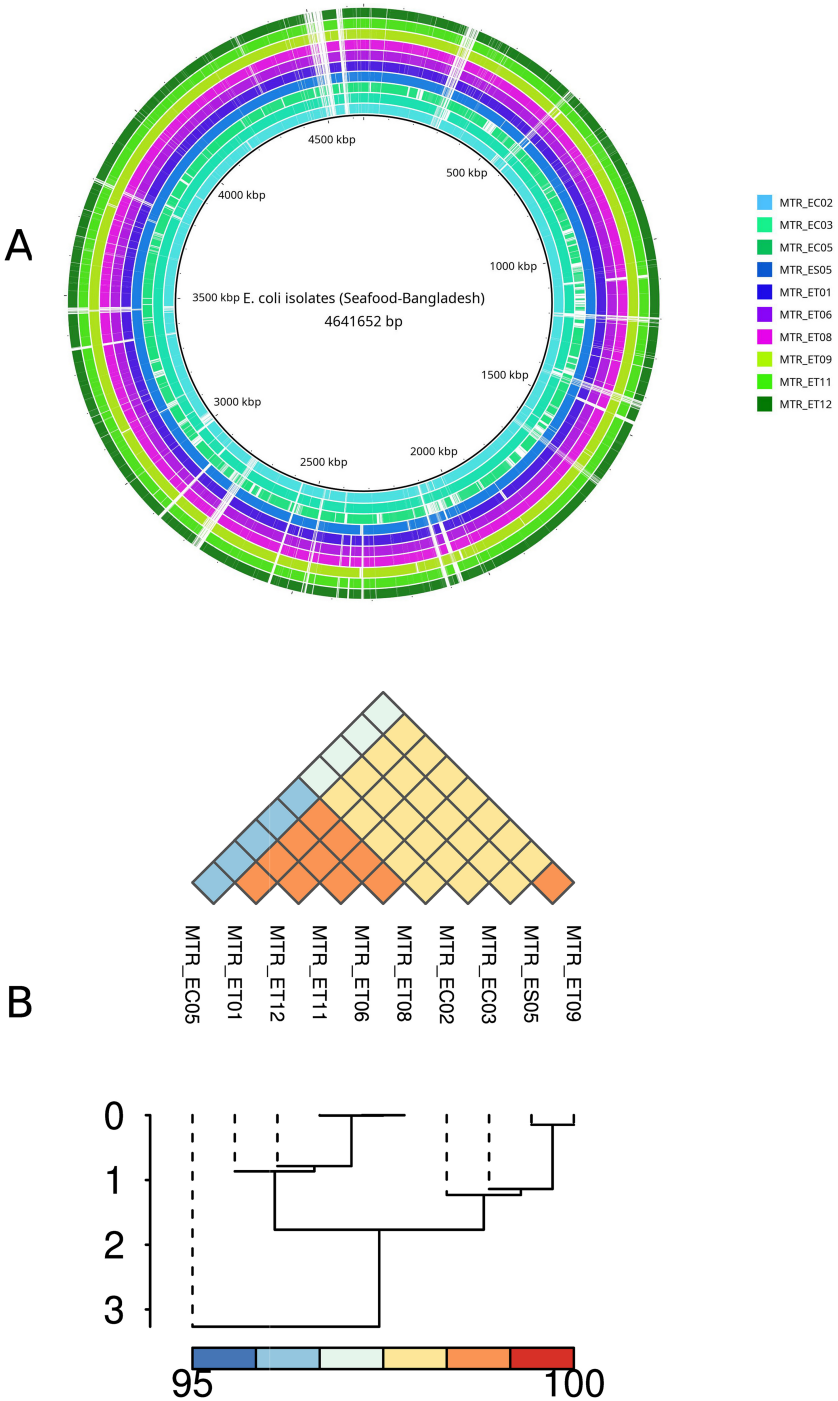
Genomic characteristics of *E. coli* isolates. **(A)** Circular genomes of the 10 isolates of *E. coli* from seafood. **(B)** ANI analysis of *E. coli* isolates. The color indicates the value of ANI; the value range is 96%–100%, with the color turning from blue to red. The numbers 0–3 refer to relative branch distance levels within the hierarchical clustering. Solid lines indicate major cluster separation, whereas dotted lines mark sub-cluster boundaries.

The genomic features of all 10 genomes were presented in the Supplementary Table S3A, with assembled chromosomes (Figure 1A), comprising a total of 4,641,652 bp. Genome sizes ranged from 4,048,090 to 4,961,915, while GC contents varied between 50.5% and 50.89%. While most isolates exhibited unique MLST and cgST profiles (Supplementary Table S3B), three isolates (MTR_ET06, MTR_ET08, and MTR_ET11) belonged to ST1431 and cgST104784. Isolates MTR_ES05 and MTR_ET09 were assigned to ST10 and a variant ST10*, respectively, but each had distinct cgSTs (27,089 and 23,638, respectively). Serotype profile of the *E. coli* isolates showed variations among seafood sources: isolates recovered from shrimp and tuna fish possessed both O and H antigens, while crab exhibited only a single antigen type, either O or H alone.

ANI values were calculated to assess the pair-wise nucleotide-level similarity among *E. coli* strains, ranging from 96 to 100% (Figure 1B). The resulting dendrogram revealed three distinct phylogenetic clades: the first clade exclusively contained tuna-derived isolates (MTR_ET11, MTR_ET06, MTR_ET08, MTR_ET12, and MTR_ET01); the second clade comprised isolates from multiple seafood sources, including tuna, shrimp, and crab (MTR_ET09, MTR_ES05, MTR_EC02, and MTR_EC03); and the third clade consisted solely of MTR_EC05, a crab isolate that formed a distinct, independent lineage.

### 3.2 Pangenomic analysis

In the pangenome analysis, the highest percentage of genes was strain-specific (56.4%), while core genes comprised 9.4% and dispensable genes 34.2% (Supplementary Figure S1 and Supplementary Tables S4A–C). The highest number of strain-specific genes was found in genomes of *E. coli* isolates in carbs, with MTR_EC05 (4246), MTR_EC05 (1484), and MTR_EC03 (244). In the isolates from shrimp, 200 specific genes were identified in MTR_ES05. Among *E. coli* from tuna, the highest number of core genes was found in MTR_ET01 (207), followed by MTR_ET12 (200), MTR_ET11 (109), MTR_ET09 (Balistreri et al., 2025), MTR_ET06 (3), and MTR_ET08 (2). However, we found partial sequence homology in core genes that were shared among 10 isolates (Supplementary Figure S2 and Supplementary Table S4D). According to the presence-absence genes matrix, MTR_ET12 and MTR_ET01, MTR_ET08 and MTR_ET06, and MTR_ET09 and MTR_ES05 were highly correlated. Due to the high number of genes in MTR_EC05, a separate denogram was generated, which differed significantly from those of other isolates (Supplementary Figure S3 and Supplementary Table S4E).

In the functional categories of Clusters of Orthologous Groups (COG), the highest number of genes was involved in metabolism, followed by poorly characterized cellular processes, signaling, and information storage and processing. In metabolism categories, genes related to inorganic ion transport and metabolism were highly prevalent in one genome (MTR_ET11) of tuna fish. In contrast, other genes were present in low percentages among all the genomes. Genes related to signal transduction mechanisms in cellular processes and signaling categories presented a higher rate than other genes involved in this process and were found to be the highest percentage in the genome of the crab (MTR_EC02). For information storage and processing, transcriptional factors were present in high numbers compared to other genes and were highly present in the shrimp genome (MTR_ES05). The highest percentage of genes involved in general function prediction only was present in the poorly characterized group and found in the MTR_ET06 and MTR_ET08 genomes present in the poorly characterized group and was found in the MTR_ET06 and MTR_ET08 genomes (Supplementary Figure S4 and Supplementary Table S4F).

### 3.3 Antimicrobial resistance genes (ARGs) in chromosomes of isolated *E. coli* from seafood

Several ARGs were identified across all *E. coli* isolates from seafish, with the highest number found in crabs. *AmpC* beta-lactamase was present in all isolates. *CTX-M-15* was found in MTR_EC03, and *bla*_DHA-1_ in MTR_EC02 (Supplementary Tables S5A,B). Moreover, fluoroquinolone resistance (*qnrS1*, *qnrB4*) in MTR_EC02, tetracycline resistance (*tetA*) in all genomes except MTR_EC02 and MTR_EC05, sulfonamide resistance (*sul1*) in MTR_EC02, aminoglycoside resistance (*cpxA*) in MTR_EC03, MTR_ET01, MTR_ET06, MTR_ET08, MTR_ET11, MTR_ET12, vancomycin resistance (*vanG*) in all genomes except MTR_EC02 and MTR_EC05 were observed.

All isolates exhibited a broader range of efflux pump systems and additional resistance mechanisms (Supplementary Table S5A), including protein overexpression and regulatory mutations (e.g., *soxR, soxS*, and *marA*), except for MTR_EC05. The major facilitator superfamily (MFS) pumps (e.g., *emrK, emrY, mdfA*, and *mdtM*), resulting in resistance to tetracyclines, phenicols, and other antibiotics, were present in almost all genomes. The genomes also included ATP-binding cassette (ABC) transporters, such as *msbA* and *TolC*, that contribute to resistance to nitroimidazoles and peptide antibiotics.

**Figure 2:**
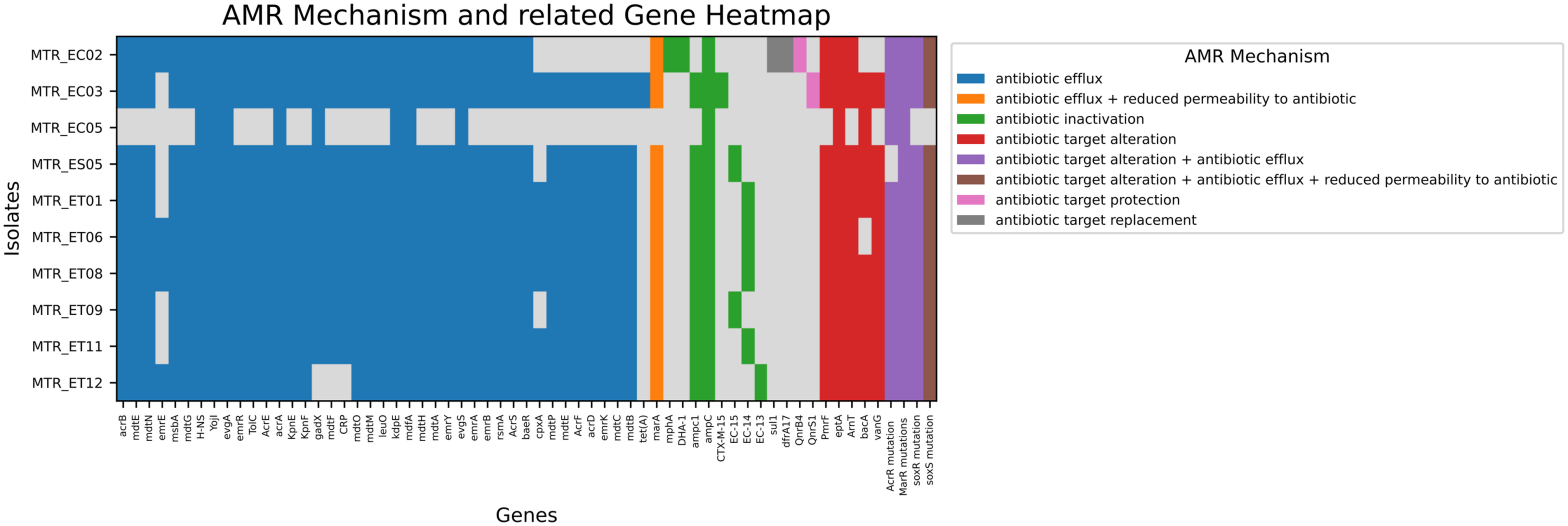
Presence of AMR genes and their resistance mechanisms in 10 *E. coli* isolates from seafood, each color corresponds to a specific AMR (antimicrobial resistance) mechanism. Blue: antibiotic efflux; Orange: antibiotic efflux + reduced permeability to antibiotic; Green: antibiotic inactivation; Red: antibiotic target alteration; Purple: antibiotic target alteration + antibiotic efflux; Pink: antibiotic target alteration + antibiotic efflux + reduced permeability to antibiotic; Dark purple: antibiotic target protection.

The distribution of resistance genes due to chromosomal mutation was: *gyrA* (MTR_ET01), *parC* (MTR_ET01), *PBP3* (All), *cyaA* (MTR_EC02), *GlpT* (except MTR_EC04, MTR_ES05, and MTR_ET09), *UhpT* (MTR_ET11), and *EF-Tu* (All). Biocide resistance and metal resistance genes were found across all genomes. We also observed the presence of additional resistance factors such as antibiotic target protection, antibiotic target alteration, antibiotic inactivation, reduced permeability to antibiotics, and other mechanisms (Figure 2). All genomes showed the concurrent presence of multiple ARGs (Supplementary Table S5A).

### 3.4 Virulence genes (VGs) in the chromosomes of isolated *E. coli* from seafood

A substantial number of VGs were found with a variation across all *E. coli* isolates, exhibiting considerable variation in their distribution patterns. Tuna-derived isolates harbored the highest abundance of adherence genes. Among those, curli fibers (*cgsD, csgA, csgB, csgB, csgC, csgD, csgF*), which play a role in biofilm formation, were detected, while MTR_EC02 contains *csgA* only, and *csgC* was only found in MTR_EC05. Nearly complete fimbrial operon (*fimA-fimI*) was identified across all genomes, except for MTR_EC02, which lacked *fimB* and *fimI*.

Multiple autotransporter genes (*tibA, tibC, aida, aatA, agn43, cah, ehaA, ehaB, air/eaeX, upaG, upaH, fepA-fepD, fepG*) were found with variable distribution patterns among the *E. coli* isolates. MTR_EC05, MTR_ES05, and MTR_ET09 possessed the most extensive repertoire of these genes. Iron uptake genes (*sitA, fes, entA-entF, entS*) were present in almost all genomes, though distribution showed strain-specific variations: *sitA* was only found in MTR_EC02, *entD* was absent in MTR_EC02 and MTR_EC05, and *entE* was missing from MTR_EC05.

**Figure 3:**
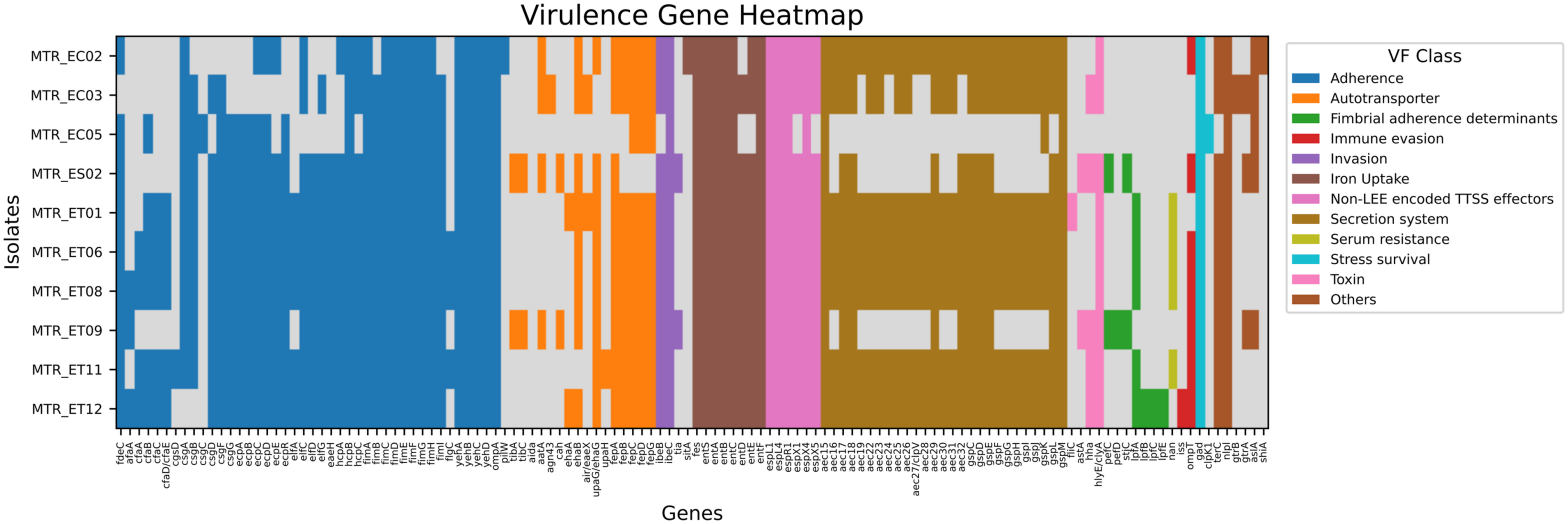
Virulence gene profile of *E. coli* isolates displayed as a heatmap. Heatmap illustrating the distribution of virulence-associated genes among the *E. coli* isolates obtained in this study. Each column represents an individual virulence gene, and each row corresponds to an isolate. The presence of a gene is indicated by a color-coded block, whereas its absence is represented in grey. Virulence genes are grouped into functional categories, with the following color representations: Adherence (blue), Autotransporter (orange), Fimbrial adherence determinants (green), Immune evasion (red), Invasion (purple), Iron uptake systems (brown), Non-LEE encoded TTSS effectors (light pink), Secretion system (olive green), Serum resistance (yellow-green), Stress survival (cyan), Toxin (pink), and Others (dark brown), as indicated in the legend. The heatmap demonstrates diversity in the virulence gene composition among isolates, highlighting variation in potential pathogenicity.

Toxin-related genes had differential distribution patterns. The hemolysin gene (*hlyE/clyA*) was present in all isolates except MTR_EC05, while *astA* was identified in MTR_ES05 and MTR_ET09. The gene, *hha* was found in MTR_EC03, MTR_ES05, MTR_ET09, MTR_ET11, and MTR_ET12, but absent in MTR_EC02, MTR_EC05, MTR_ET01, MTR_ET06 and MTR_ET08. Stress survival genes were ubiquitously distributed, with *gad* in all isolates, while *clpK1* was uniquely detected in MTR_EC05 (Figure 3). Other genes related to virulence were also detected and summarized in Supplementary Table S5C.

### 3.5 Genomic islands and prophage-associated resistance and virulence determinants

A variable number of genomic islands and prophages were identified in the genomes of *E. coli* isolated from crabs, including some notable ARGs, such as *blaDHA-1, blaCTX-M-15, sul1, tetA*, and *mph(A),* which confer resistance to beta-lactamase, sulfonamide, tetracycline, and macrolides, respectively. Several essential VGs were also detected in these GIs, with curli fiber genes (*csgC-csgG*) being particularly prominent. Additional virulence factors identified within these GIs included diverse fimbrial genes (*fimB, fimD, fimE, fimH*), adhesion factors (*fdeC*), secretion system proteins (*espX4, rhs, vgrG, espL1, vgrG, tssM, tssA, hcp1*), and immune evasion genes (*gmhA* and *gtrB*).

*Escherichia coli* derived from shrimp and tuna fish also exhibited a large number of GIs and prophages harboring ARGs related to multidrug efflux pump (*mdfA*) and several virulence factors (Supplementary Table S6).

### 3.6 Mobile elements and plasmids identified from *E. coli* isolated in this study

Plasmids were detected in only three of the 10 *E. coli* genomes, all exclusively from crab isolates. MTR_EC02 harbored plasmids of multiple plasmid replicon types, including IncFII, IncFIA, IncFIB (pHCM2), and ColRNAI. IncFII carried ARGs [*qnrB4, dfrA17, qacE, mph(A), sul1, bla*_DHA-1_] associated with IS*6100*. No VGs were found in these plasmids. MTR_EC03 and MTR_EC05 contained only the Col440I plasmid, which harbored no ARGs or VGs.

Across all 10 genomes, VGs were consistently identified in chromosomes flanked by mobile elements, whereas ARGs were located in both chromosomes and plasmids (Supplementary Table S7). Genes involved in Integration/Excision, Replication / Recombination / Repair, Phage, Transfer, and stability genes were detected in both the chromosome and plasmid of all isolates (Supplementary Table S5D).

### 3.7 Population structure and its distribution

**Figure 4:**
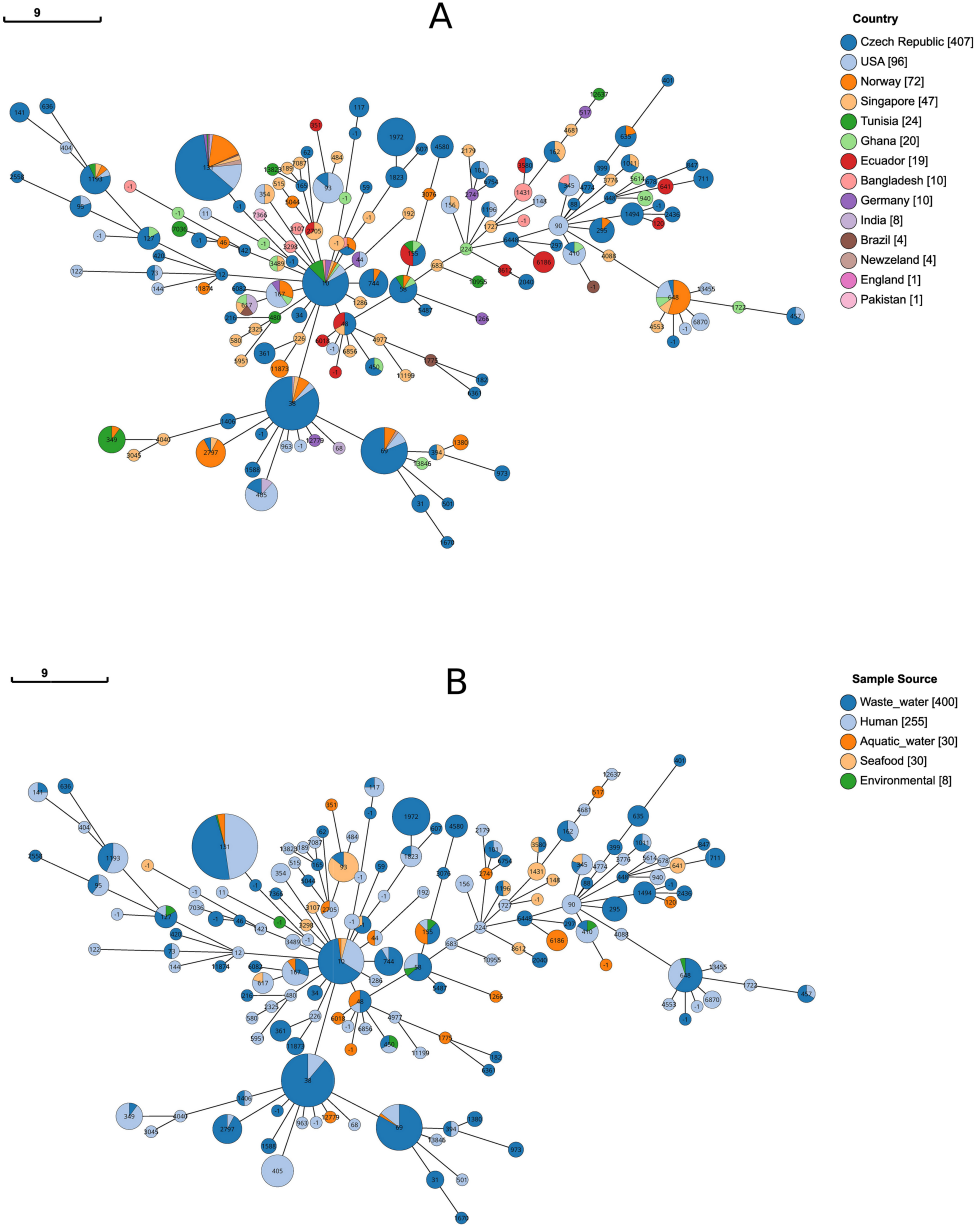
Population structures and their distribution. **(A)** Population structure of different sequence types among 723 strains from around the globe, by country **(B)** Population structure of varying sequence types of 723 strains from around the world according to their sample source.

A total of 102 sequence types (STs) were identified among the 723 isolates, including five STs (ST3107, ST3298, ST345, ST1431, ST10) from our isolates (Supplementary Table S2). Genomic similarity was observed between *E. coli* belonged to ST345 and ST10 in this study and the isolates reported from Czech Republic (Wastewater) and USA (Human) and the isolates from Czech Republic (Wastewater and Human), Singapore (Human), Germany (Seafood and Aquatic water), Norway (Wastewater), USA (Human), Tunisia (Human), and Ghana (Human), accordingly (Figure 4A). *E. coli* ST27089 and ST23638 exhibited limited geographical distribution. Additionally, a few other STs were more prevalent in the comparison strains, including ST131 was the highest occurrence of a single type of ST detected from the Czech Republic (Wastewater, Human), New Zealand (Aquatic water), Norway (Wastewater), USA (Human), and Singapore (Human), and ST38 in Czech Republic (Wastewater and Human), Norway (Wastewater), India (Human), and USA (Human) (Figure 4B).

### 3.8 Global phylogenetic relationships and comparative insights

**Figure 5:**
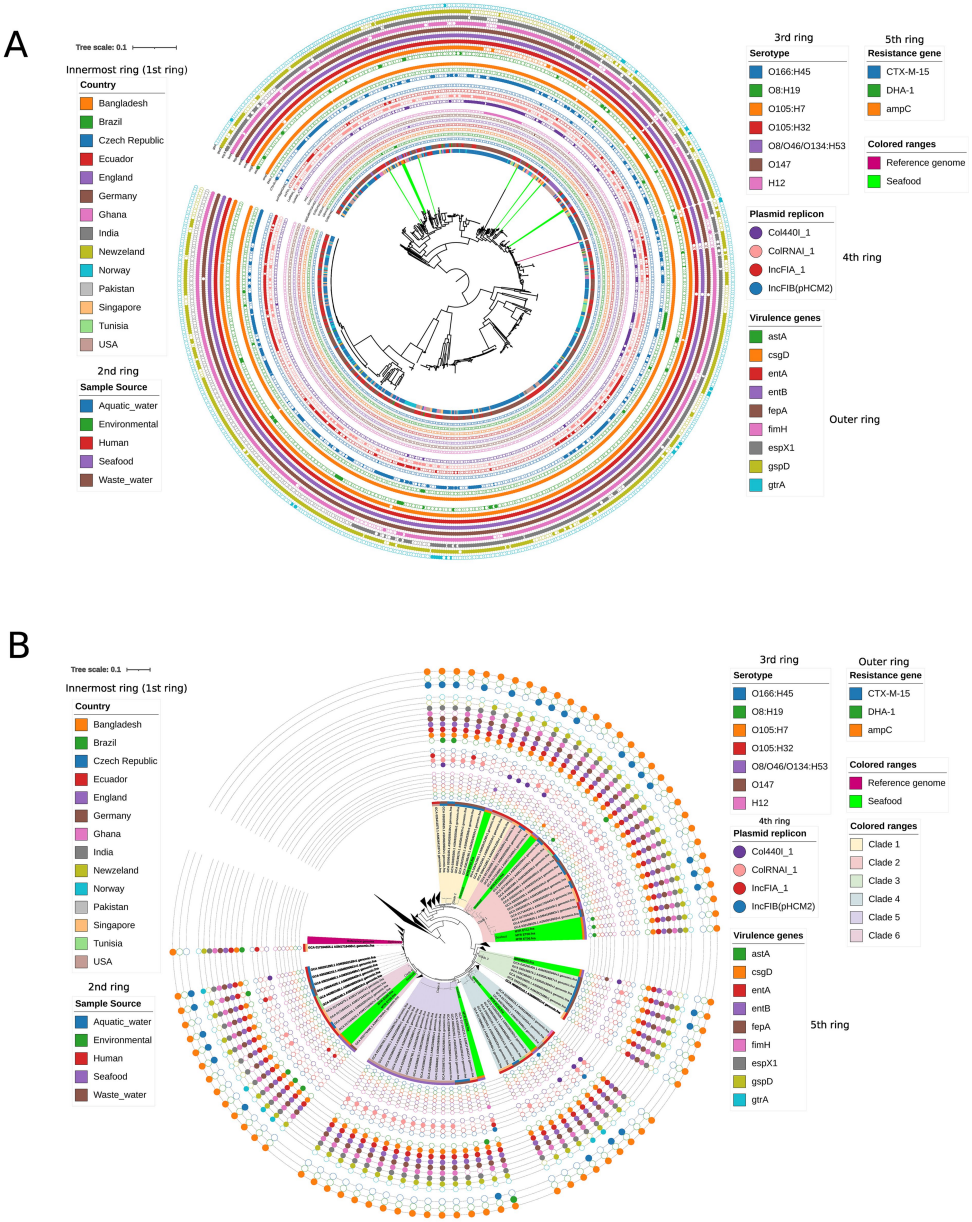
Global phylogeny and zoomed-in clade of 724 *E. coli* genomes. **(A)** Shows the maximum-likelihood global phylogeny constructed from 724 genomes, including the reference genome and 10 seafood-derived isolates analyzed in this study (nine additional genomes were excluded from assembled genomes due to their poor sizes were outside the expected range, indicating likely sequencing or assembly errors during Parsnp v2.0 filtering). A total of 108,456 core SNPs revealed diverse phylogenetic clustering, with concentric metadata rings representing country (*n* = 1), the most inner circle, followed by sample source (2nd ring, *n* = 1), serotype (3rd ring, *n* = 6), plasmid replicons (4th ring, *n* = 4), resistance genes (5th ring, *n* = 3), and virulence genes (6th ring, *n* = 9). The reference genome (pink) and the 10 isolates (green) are prominently highlighted in the inner tree. **(B)** Resents a zoomed-in view of the specific clade containing these 10 isolates, allowing clear visualization of their immediate phylogenetic neighbors and cluster relationships using the same metadata annotations as in the global tree. All the rings are the same as **(A)**, except for virulence genes (5th ring, *n* = 9) and resistance genes (6th ring, *n* = 3).

The genomes of 10 *E. coli* were compared with a total of 713 global isolates, including a reference genome from different sources in different countries, using core genome SNP analysis (Supplementary Table S8). According to phylogenetic analysis, strains from this study form several minor clusters with other global strains (Figure 5A). A total of 108,456 SNPs were observed following core genome alignment, resulting in diverse phylogenetic clustering. Based on SNP tree analysis, isolates from tuna fish (MTR_ET06, MTR_ET0, and MTR_ET11) formed a close cluster with the isolates from diverse sources from different countries, such as Ecuador (seafood) and the Czech Republic (wastewater). MTR_ET01 formed a cluster with isolates from the USA (Human), the Czech Republic (wastewater), and MTR_ET12, which formed a cluster with isolates from Singapore (Human). The isolates from one tuna fish and shrimp (MTR_ET09 and MTR_ES05) showed a closer cluster with the isolates from Singapore (Human), Germany (Seafood), and the Czech Republic (Human). However, slightly different branches were observed for the isolates from crab, showing MTR_EC02 with Czech Republic (Wastewater), Singapore (Human), and USA (Seafood), and MTR_EC03 with Pakistan (Wastewater). Additionally, MTR_EC05 exhibited a distinct node within some closely clustered regions (Figure 5B).

Serotype comparison revealed limited concordance with international strains. Only two serotypes identified in our study (08: H19 and H12) matched the isolates from the Czech Republic (wastewater), while the remaining serotypes were unique to our seafood isolates (Figure 5A).

Resistance gene profiling revealed that *ampC* was nearly ubiquitous, present in approximately 100% of isolates. The *bla*_CTX-M-15_, identified only in MTR_EC02, had been distributed among isolates from geographically diverse human and environmental sources, including the Czech Republic (wastewater and human), India (Human), the USA (Human), Tunisia (Human), and Norway (Wastewater). The resistance gene, *bla*_DHA-1_, found also in MTR_EC02, appeared to be dominant in isolates from Norway (Wastewater) and Tunisia (Human) (Figure 5A).

The essential VGs involved in key pathogenic mechanisms, including adhesion (*fimH*), toxin production (*astA*), biofilm formation (*csgD*), iron acquisition (*entA, entB, fepA*), secretion system (*espx1, gspD*), and surface structure modulation (*gtrA*) were found to be distributed in global strains. Isolates from Tunisia (Human), Norway (Wastewater), Ghana (Environment and Human), and Singapore (Human) showed higher abundance of VGs than Bangladeshi seafood isolates characterized in this study (Figure 5A).

The Col440I_1 plasmid type, identified in MTR_EC03 and MTR_EC05, was most frequently detected in isolates from the Czech Republic (wastewater). The ColRNAI_1 and IncFIA_1 plasmids present in MTR_EC02 demonstrated broader geographic distribution, predominantly occurring in isolates from the Czech Republic (wastewater and human), the USA (Seafood and Human), and Ghana (Environmental and Human). The IncFIB (pHCM2) plasmid exhibited a more restricted distribution, being associated with the Czech Republic isolates (wastewater) (Figure 5A).

## 4 Discussion

This comprehensive genomic study investigates ESBL-producing *E. coli* from seafood in Bangladesh. *E. coli* is noted for its genetic flexibility and its ability to transmit ARGs and VGs through horizontal gene transfer (Li et al., 2024; Hasegawa et al., 2018). This analysis offers critical insights into the landscape of AMR and virulence among these pathogens, while also contextualizing their relationship with global strains. The findings underscore significant public health and food safety concerns, as well as the broader challenges associated with the dissemination of AMR within aquatic food systems.

In Bangladesh, untreated sewage, agricultural runoff, and livestock waste consistently introduce *E. coli* and other faecal bacteria into aquaculture and coastal waters, with monsoon-driven hydrological pulses further intensifying microbial loads. Coupled with unsafe wash water, inadequate chilling, and frequent cross-contamination during harvest, processing, and retail, these conditions enable persistent transfer of faecal bacteria onto processed food, exposing a major weakness in the national supply chain (Parvin et al., 2022). The ANI-based phylogenetic analysis in this study (Figure 1B), revealing source-specific clustering, particularly the segregation of tuna isolates into a distinct clade sharing identical STs (ST1431/cgST104784), indicates possible vertical transmission within aquaculture systems or persistent contamination from shared sources throughout the seafood production chain. The presence of MTR_EC05 as a phylogenetically distant outlier suggests introduction from an alternative contamination source, highlighting the complexity of microbial ecology in seafood supply chains (Sheng and Wang, 2021). Decentralized wastewater treatment, improved runoff control, safe-water access, and stricter hygiene and cold-chain management across aquaculture and food-handling nodes provide practical, locally actionable means to prevent faecal bacterial contamination in Bangladesh (Islam and Islam, 2022).

The serotype variation between seafood sources, complete O: H antigen profiles in shrimp and tuna versus incomplete antigen expression in crab, may reflect differential immune evasion strategies or adaptation to distinct ecological niches (Rheman et al., 2024). Functional genomic characterization revealed adaptive features directly relevant to seafood contamination dynamics across all sources. The significant presence of inorganic ion transport and metabolism genes in tuna isolates (Figure 3), the high prevalence of signal transduction genes in crab isolates, and the abundance of transcriptional factors in the shrimp isolate collectively demonstrate that seafood-associated *E. coli* populations harbor genetic architectures specifically adapted for persistence under refrigeration and processing conditions, tolerance to preservation-associated stresses, and expression of virulence determinants that enhance pathogenic potential (Sung et al., 2024; Xie et al., 2022).

Core genome SNP analysis, revealing 108,456 polymorphisms, demonstrates substantial evolutionary divergence among the 723 isolates, yet the phylogenetic clustering of Bangladeshi seafood isolates belonged to ST345 and ST10 with international strains from Ecuador, the Czech Republic, the USA, Singapore, Germany, and Pakistan across human clinical, wastewater, and seafood sources indicates recent common ancestry and ongoing global dissemination of this lineage (Figure 5A). Globally, ST10 is recognised as a “high-risk” and frequently observed sequence type in extraintestinal clinical *E. coli* infections, including urinary tract infections, bloodstream infections, and other hospital/community-acquired infections, and is often associated with antimicrobial resistance (Manges et al., 2019). According to Ecuador’s Aquaculture and Fisheries Industry, Ecuador represents a major seafood exporter. The phylogenetic linkages with Bangladeshi tuna isolates (MTR_ET06, MTR_ET08, MTR_ET11) and strains from Ecuadorian seafood and Czech wastewater are particularly significant for food safety, suggesting potential contamination through international seafood trade networks or shared environmental reservoirs affecting geographically distant aquaculture operations (Alexandre et al., 2025).

The phylogenetic clustering of crab isolates to human clinical strains from Singapore and the USA, alongside wastewater isolates from the Czech Republic and Pakistan, suggests bidirectional transmission between environmental, food, and human (Figure 5A). This interconnectedness indicates that contaminated seafood may serve as a vehicle for introducing antimicrobial-resistant strains into human populations, while simultaneously, wastewater contamination can introduce human-associated pathogens back into aquatic food production environments, creating a concerning epidemiological cycle (Meradji et al., 2025; Albini et al., 2022).

The limited serotype concordance with international strains, only O8: H19 and H12 matching Czech wastewater isolates (Figure 5A), while other serotypes remain unique to Bangladeshi seafood, suggests that, despite core genome relatedness, surface antigen diversity has evolved through localized selection pressures or recombination events (Liu et al., 2020; Lang et al., 2023). This serotype divergence has important implications for surveillance and outbreak detection, as traditional serotype-based monitoring may fail to identify genetically related strains that have undergone antigenic variation. The unique serotypes in Bangladeshi seafood could represent endemic adaptations to local aquaculture environments and emerging variants with potential to disseminate internationally through seafood trade (Kavinesan et al., 2023).

Consistent with global ESBL-producing *E. coli*, seafood-derived isolates in this study harbored chromosomal *AmpC β*-lactamase, conferring broad-spectrum resistance to β-lactams, including third-generation cephalosporins and β-lactamase inhibitor combinations (Figure 5A). Phenotypic AST showed uniform resistance to third-generation cephalosporins (cefotaxime and ceftazidime) across all isolates, and this was strongly aligned with the genomic resistome (Supplementary Table S5B). All isolates carried a plasmid-mediated *bla-ampC* gene, which is known to confer resistance to expanded-spectrum cephalosporins and is not inhibited by β-lactamase inhibitors (Thomson, 2010; Jacoby, 2009). In contrast, blaCTX-M-15 and blaDHA1 were detected only in a minority of isolates, indicating that *ampC* production was the principal mechanism explaining the observed cephalosporin-resistance phenotype. The absence of *blaCTX-M-15* and *blaDHA1* in most isolates suggests that mechanisms such as *ampC* hyperproduction, efflux pumps, or porin alterations may contribute to the observed resistance phenotype (Li et al., 2015). These findings are consistent with global reports on the increasing clinical relevance of plasmid-borne *ampC* enzymes in *E. coli* (Philippon et al., 2002; Tamma and Rodriguez-Bano, 2017). This ubiquitous distribution across environmental, food production, and clinical pathways presents serious public health challenges by substantially limiting treatment options for infections originating from contaminated seafood consumption (Farrukh et al., 2025). Chromosomal mutations affecting antibiotic targets, *gyrA, parC, PBP3, and EF-Tu*, constitute stable, vertically inherited resistance mechanisms that persist regardless of plasmid loss or mobile element excision (Figure 2) (Bush et al., 2020; Darby et al., 2023; Long et al., 2025). Their widespread occurrence across Bangladeshi seafood isolates indicates sustained antimicrobial selection pressure within aquaculture environments, evidencing systematic antibiotic misuse throughout production chains.

The extensive efflux pump repertoire generates constitutively resistant phenotypes that persist independently of antibiotic exposure by continuously reducing intracellular antimicrobial concentrations (Zhang et al., 2024). These systems confer cross-resistance to structurally unrelated antimicrobials and chemical disinfectants used in processing facilities. Consequently, bacteria with robust efflux systems can survive sanitation procedures, persist on food contact surfaces, and contaminate finished products.

Pangenome analysis revealed that strain-specific genes (56.4%) substantially outnumber core genes (9.4%), demonstrating remarkable genomic plasticity within seafood-associated *E. coli* populations (Supplementary Figure S1). Comprehensive ARGs and VGs profiling also revealed substantial genetic diversity across seafood-derived *E. coli* isolates. This genetic flexibility indicates active horizontal gene transfer and adaptive evolution driven by antimicrobial pressures and environmental stresses encountered throughout aquaculture production, processing, and storage. The chromosomal localization of typically plasmid-borne resistance genes, including *bla*_CTX-M-15_, *qnrS1, qnrB4, tetA, and sul1,* and chromosomally encoded virulence genes flanked by mobile genetic elements, provides evidence of mobile element-mediated genomic restructuring (Supplementary Table S7). Such chromosomal incorporation stabilizes resistance and virulence determinants by preventing gene loss during bacterial replication in the absence of selection pressure, thereby establishing permanently resistant and pathogenic strains that persist within seafood products (Chekole et al., 2025). Interestingly, the variable distribution of curli components across isolates, with MTR_EC02 possessing only *csgA* and *csgC* exclusively present in MTR_EC05 (Supplementary Table S6), suggests strain-specific biofilm-forming capabilities that may influence persistence characteristics within different seafood matrices or processing environments (Elafify et al., 2024; Balistreri et al., 2025).

Particularly concerning is the co-localization of ESBL genes (*bla*_DHA-1_, *bla*_CTX-M-15_) with VGs (e.g., curli operons, fimbrial genes, type VI secretion system components, and immune evasion factors) within mobile genetic islands and prophages in crab-derived isolates (Supplementary Table S6). This genetic architecture facilitates the simultaneous horizontal transfer of both pathogenic and resistance traits (Tokuda and Shintani, 2024). The identification of resistance genes (*qnrB4, dfrA17, sul1, bla*_DHA-1_) associated with IS*6100* on IncFII plasmids of a seafood-associated *E. coli* further demonstrates active horizontal transfer capability among these populations (Supplementary Table S7). IS*6100*, recognized as a highly mobile IS, facilitates both intra- and inter-genomic rearrangements (Varani et al., 2021), accelerating resistance evolution and enabling rapid bacterial adaptation to antimicrobial environments encountered during seafood production and processing.

The observation that international isolates from Tunisia, Norway, Ghana, and Singapore exhibit higher virulence gene abundance compared to Bangladeshi seafood strains suggests geographic variation in pathogenic potential, possibly reflecting differences in aquaculture practices, environmental conditions, or evolutionary histories (Figure 5A) (Prakasan et al., 2022). However, the presence of core virulence determinants in Bangladeshi isolates (Figure 3) indicates sufficient potential pathogenic capacity to cause foodborne illness if not cooked or handled properly, and the concurrent presence of resistance determinants compromises treatment options.

Our findings indicate that seafood products represent high-risk vehicles for transmitting antimicrobial-resistant *E.coli*. There is an urgent need for stringent microbiological surveillance throughout seafood production chains, implementation of validated pathogen reduction technologies, elimination of prophylactic antibiotic use in aquaculture, enhanced wastewater treatment to prevent environmental contamination, and harmonized international food safety standards to protect consumer health and maintain the integrity of global seafood trade. Without coordinated One Health interventions addressing the aquaculture-environment-human interface, seafood will continue serving as a potentially important reservoir and dissemination pathway for antimicrobial-resistant pathogens, undermining global efforts to combat the AMR crisis while compromising food security and public health.

## 5 Conclusion

This study provides the first comprehensive genomic characterization of ESBL-producing *E. coli* from seafood in Bangladesh, revealing significant genetic diversity, multidrug resistance, and the presence of key virulence factors. The detection of diverse MLSTs, *β*-lactamase genes such as *blaCTX-M-15, ampC,* and *blaDHA-1*, and plasmid-mediated ARGs underscores the potential of seafood to act as a reservoir and transmission vehicle for AMR. Notably, crab isolates harbored the highest number of ARGs and plasmids, while virulence genes were widely distributed across isolates, suggesting possible pathogenic potential. Comparative genomic analysis demonstrated strong links between Bangladeshi isolates and global strains from humans, animals, and the environment, highlighting the interconnectedness of AMR dissemination through food systems and aquatic environments. These findings emphasize the urgent need for strengthened AMR surveillance and strict hygiene practices in aquaculture and seafood processing, guided by a One Health approach, to mitigate public health risks associated with seafood-borne *E. coli.*

## Data Availability

The datasets presented in this study can be found in online repositories. The names of the repository/repositories and accession number(s) can be found in the article/Supplementary material.

## Footnotes

1
https://www.arc.ox.ac.uk

2
https://github.com/tseemann/mlst

3
https://cge.food.dtu.dk/services/MLST/

4
https://cge.food.dtu.dk/services/cgMLSTFinder/

5
https://www.genomicepidemiology.org/services/

6
https://github.com/tseemann/abricate

8
https://cge.food.dtu.dk/services/MobileElementFinder/

9
https://proksee.ca/

10
https://ecotyper.stanford.edu

11
https://cge.food.dtu.dk/services/SerotypeFinder/

## Supplementary material

SUPPLEMENTARY FIGURE S1 The percentage of genes distributed among the ten isolates in the Pangenomic analysis.

SUPPLEMENTARY FIGURE S2 UpSet plot showing shared and unique gene intersections among the analyzed genomes. The upper histogram displays the number of genes contained in each intersection (i.e., gene sets shared by a specific combination of genomes). Each bar corresponds to the intersection highlighted directly below it in the connected-dot matrix. Taller bars represent larger shared gene sets. The dot matrix indicates which genomes contribute to each intersection: filled circles denote presence in a genome, and connected lines show the combination of genomes forming that intersection. The left bar plot shows the total number of genes identified in each genome.

SUPPLEMENTARY FIGURE S3 Presence-absence matrix of gene cluster among the ten *E. coli* isolates.

SUPPLEMENTARY FIGURE S4 Distribution of COG functional categories among the ten *E.coli* isolates.

SUPPLEMENTARY TABLE S1 List of 722 *E. coli* strains selected through two independent PubMed queries based on title, full-text availability, relevance to the study, and availability of assembled genomes. The table includes all publications identified through the searches and metadata, along with the corresponding genomes downloaded from NCBI. Multi-sheet table summarizing the PubMed search strategy, metadata, and genome selection process for this study. **(A)** (First_query) contains the full search string and results from the initial query, while **(B)** (Metadata_first_query) provides metadata for genomes retrieved from that query. **(C)** (Second_query) presents the search string and results from the secondary query focused on seafood-associated *E. coli*, and **(D)** (Metadata_second_query) includes the corresponding genome metadata. **(E)** (Total_no_genome_by_sample) summarizes the total number of genomes grouped by sample source across both queries.

SUPPLEMENTARY TABLE S2 Metadata for the 723 *E. coli* genomes used for minimal spanning tree analysis, including genome ID, sequence type (ST), country of origin, and sample source.

SUPPLEMENTARY TABLE S3 Summary of genomic and typing information for all genomes analyzed. **(A)** Genomic features, and **(B)** MLST and cgST profiles.

SUPPLEMENTARY TABLE S4 Pangenome analysis. **(A)** List of strain specific clusters with functional annotation and COG categories. **(B)** List of core gene clusters with functional annotation and COG categories. **(C)** List of dispensary gene clusters with functional annotation and COG categories. **(D)** Presence–absence matrix of gene clusters across 10 genomes. **(E)** Clustered accessory gene presence/absence matrix for the 10 genomes (1 = present, 0 = absent). **(F)** COG assignment of each gene cluster.

SUPPLEMENTARY TABLE S5 Summarization of ARGs **(A)**, phenotype and genotypic profile **(B)**, VGs **(C)**, and MGEs **(D)** of ten *E. coli* isolates in this study.

SUPPLEMENTARY TABLE S6 Genomic characterization of *E. coli* isolates, detailing the presence of genomic islands (GIs), prophages, virulence genes (VGs), and antimicrobial resistance genes (ARGs) within the chromosome. For each isolate, the table reports the number of GIs and prophages, the virulence factor classes and specific virulence genes identified, as well as the drug classes and corresponding ARGs detected.

SUPPLEMENTARY TABLE S7 Summary of mobile genetic elements and plasmid content in ten *E. coli* strains. The table reports detected mobile elements, plasmid types, and the presence of associated antimicrobial resistance genes (ARGs) and virulence genes (VGs).

SUPPLEMENTARY TABLE S8 Metadata and genomic characteristics of *E. coli* isolates (*n* = 723) used to construct the global phylogeny. The table includes isolate ID, sample source, and country of origin, along with detected antimicrobial resistance (AMR) genes, virulence-associated genes, plasmid replicon types, and serotypes, all of which were used to annotate and interpret phylogenetic relationships. The last 10 isolates at the bottom of the sheet were our isolates, which were compared (Yellow marking).
