# Supplementary figures and images for: Genome-wide analysis of extended-spectrum beta-lactamase-producing Escherichia coli from seafood in Bangladesh: population structure, resistome, virulome, and global dissemination patterns
Source: Front Microbiol. 2026 Feb 6;17:1737712. doi: 10.3389/fmicb.2026.1737712 (PMC12920492; doi:10.3389/fmicb.2026.1737712)

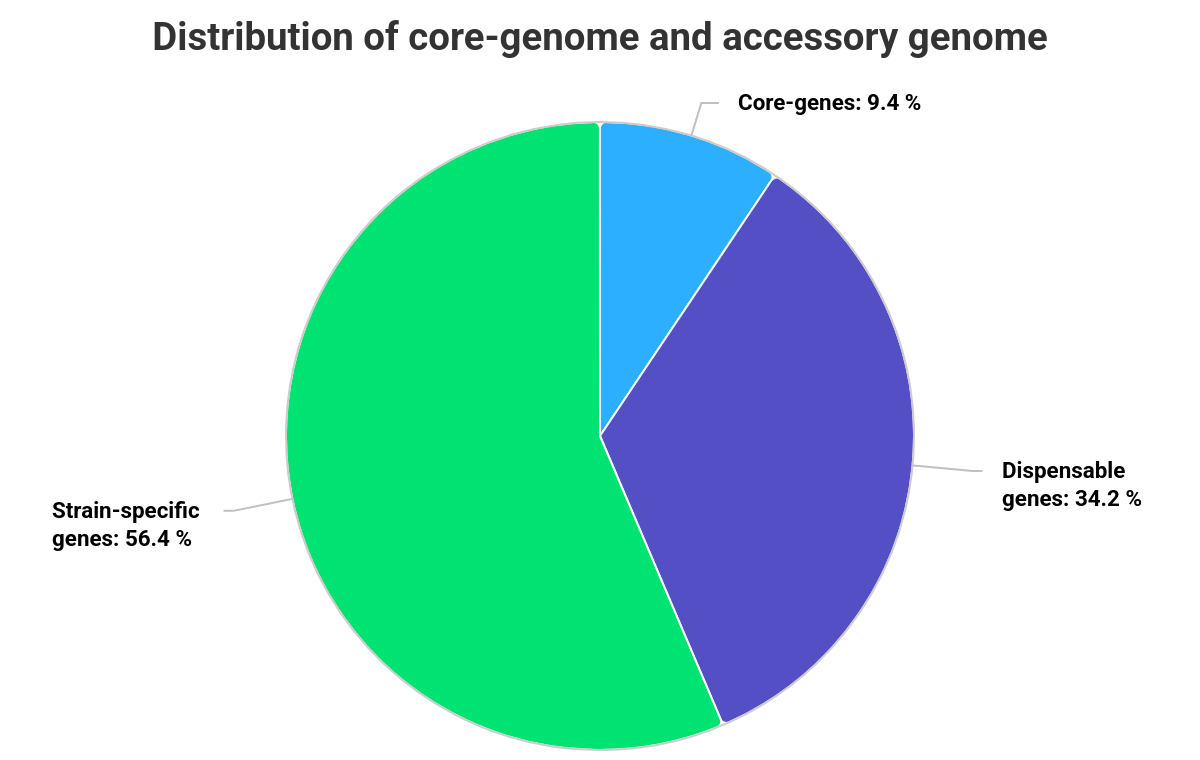

Supplement: SUPPLEMENTARY FIGURE S1 — The percentage of genes distributed among the ten isolates in the Pangenomic analysis. [file Image_1.png]

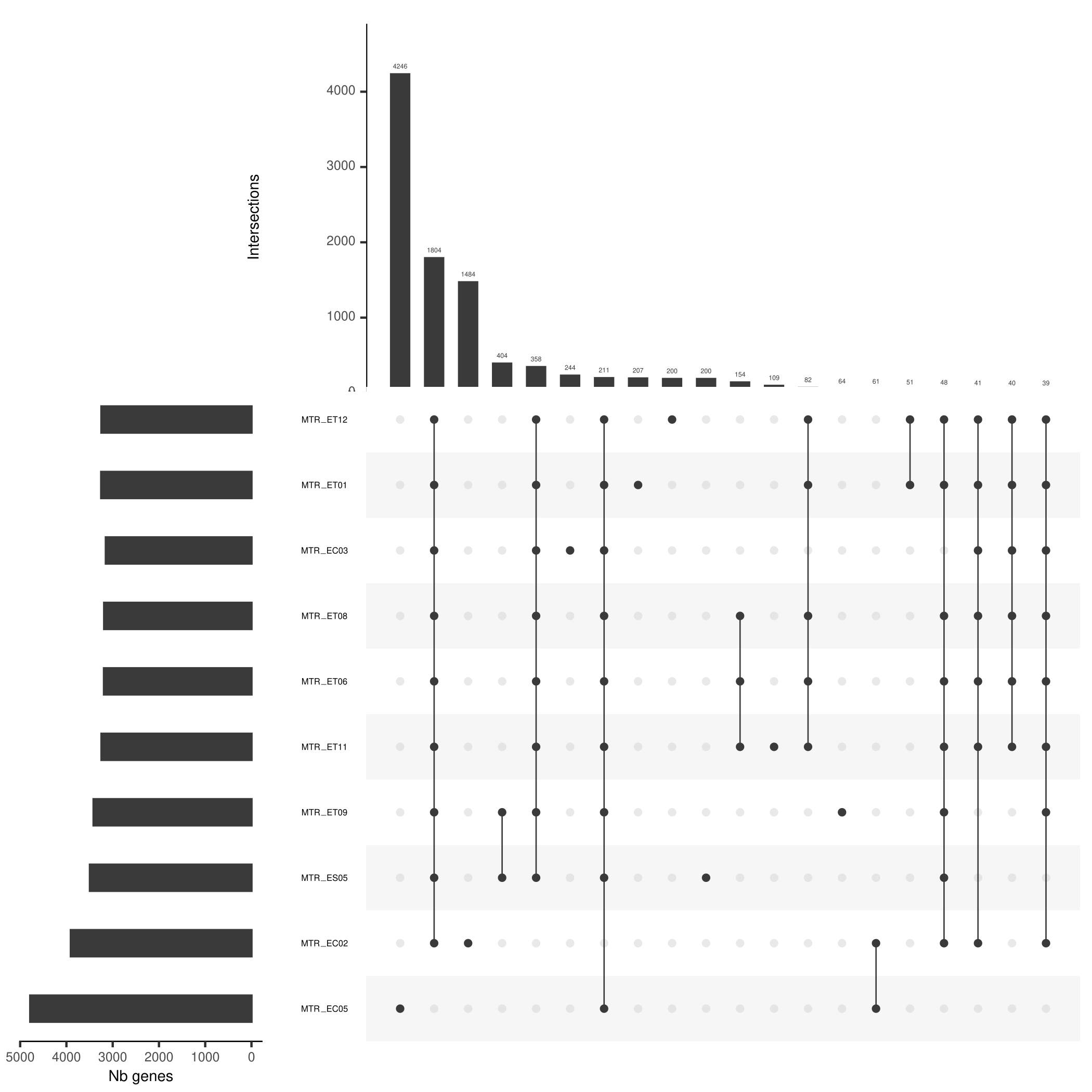

Supplement: SUPPLEMENTARY FIGURE S2 — UpSet plot showing shared and unique gene intersections among the analyzed genomes. The upper histogram displays the number of genes contained in each intersection (i.e., gene sets shared by a specific combination of genomes). Each bar corresponds to the intersection highlighted directly below it in the connected-dot matrix. Taller bars represent larger shared gene sets. The dot matrix indicates which genomes contribute to each intersection: filled circles denote presence in a genome, and connected lines show the combination of genomes forming that intersection. The left bar plot shows the total number of genes identified in each genome. [file Image_2.jpeg]

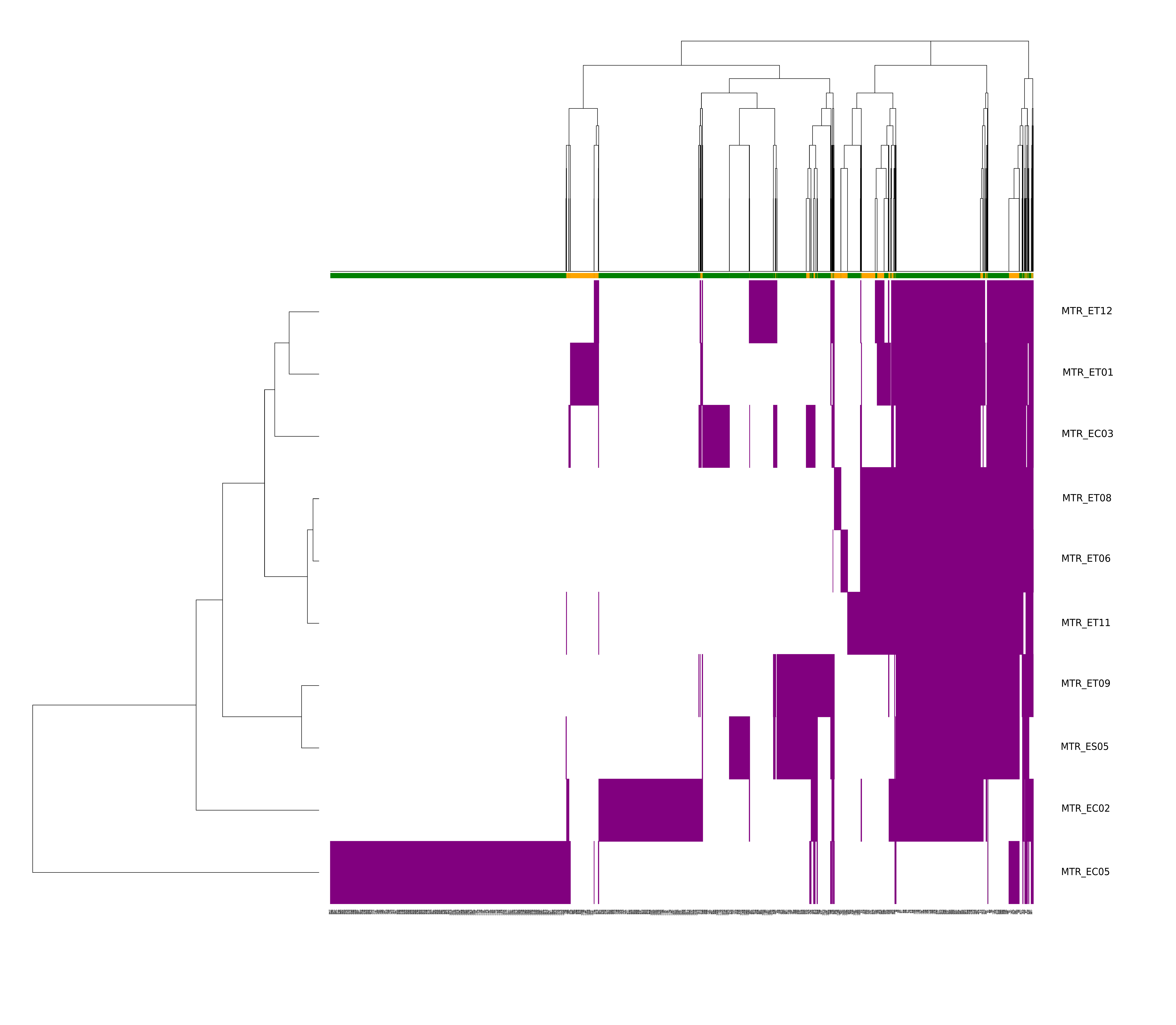

Supplement: SUPPLEMENTARY FIGURE S3 — Presence-absence matrix of gene cluster among the ten E. coli isolates. [file Image_3.jpeg]

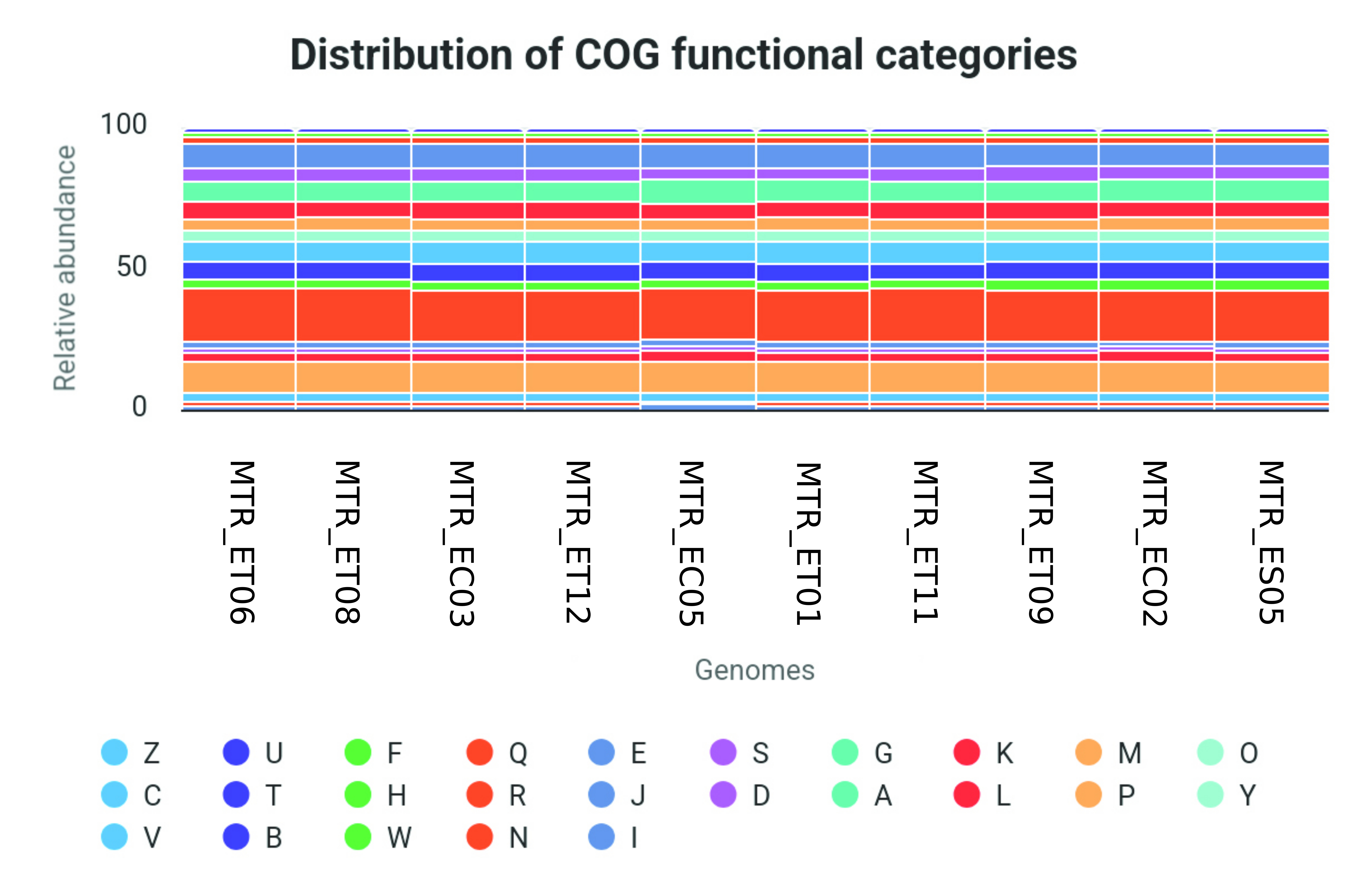

Supplement: SUPPLEMENTARY FIGURE S4 — Distribution of COG functional categories among the ten E.coli isolates. [file Image_4.jpeg]
