# Supplementary material for: Genome-wide analysis of extended-spectrum beta-lactamase-producing Escherichia coli from seafood in Bangladesh: population structure, resistome, virulome, and global dissemination patterns
Source: Front Microbiol. 2026 Feb 6;17:1737712. doi: 10.3389/fmicb.2026.1737712 (PMC12920492; doi:10.3389/fmicb.2026.1737712)
Supplement: SUPPLEMENTARY TABLE S6 — Genomic characterization of E. coli isolates, detailing the presence of genomic islands (GIs), prophages, virulence genes (VGs), and antimicrobial resistance genes (ARGs) within the chromosome. For each isolate, the table reports the number of GIs and prophages, the virulence factor classes and specific virulence genes identified, as well as the drug classes and corresponding ARGs detected. [file Table_6.docx]

**6Supplementary Table 6:** Genomic characterization of Escherichia coli isolates, detailing the presence of genomic islands (GIs), prophages, virulence genes (VGs), and antimicrobial resistance genes (ARGs) within the chromosome. For each isolate, the table reports the numbers of GIs and prophages, the virulence factor classes and specific virulence genes identified, and the drug classes and corresponding ARGs detected.

| **Isolates** | **Number of GIs** | **Number of Prophages** | **VGs** | | | **ARGs** | |
| --- | --- | --- | --- | --- | --- | --- | --- |
|  |  |  | **VF class** | **VF factors** | **VGs** | **Drug Class** | **ARGs** |
| MTR_EC02 | 10 | 8 | Adherence | Type I fimbriae | *fimB, fimE* | Aldehydes | *form(A)* |
|  |  |  |  | Curli fibers | *csgA, csgB, csgC, csgD, csgE, csgF, csgG* | Peroxides | *sitABCD* |
|  |  |  |  | *E. coli* common pilus (ECP) | *ecpR* | Macrolides | *mph(A), mdf(A)* |
|  |  |  |  | Adhesin | *fdeC* | Sulphonamides | *sul1* |
|  |  |  | Secretion system protein | Non-LEE encoded TTSS effectors | *espX4* | Beta-lactams | *bla*_DHA-1_ |
|  |  |  |  |  |  | Fluoroquinolones | *qnrB4* |
|  |  |  |  |  |  | Folates, pathways & antagonists | *dfrA17* |
| MTR_EC03 | 16 | 6 | Adherence | Glucosephosphate uridylyltransferase | *galU* | Aldehydes | *form(A)* |
|  |  |  |  | Type I fimbriae | *fimB, fimE, fimH, fimD* | Macrolides | *mdf(A)* |
|  |  |  |  | Curli fibers | *csgB, csgD, csgE, csgF, csgG* | Beta-lactams | *bla*_CTX-M-15_ |
|  |  |  | Secretion system protein | Non-LEE encoded TTSS effectors | *espX4, espL1* | Tetracyclines | *tet(A)* |
|  |  |  |  |  |  | Fluoroquinolones | *qnrS1* |
|  |  |  |  | Type VI secretion system proteins/ effectors | *rhs, vgrG* |  |  |
|  |  |  | Immune evasion | Phosphoheptose isomerase | *gmhA* |  |  |
|  |  |  |  | Bactoprenol glucosyl transferase | *gtrB* |  |  |
| MTR_EC05 | 12 | 5 | Secretion system protein | Non-LEE encoded TTSS effectors | *espX4, espL1* | Macrolides | *mdf(A)* |
|  |  |  |  |  |  | Aldehydes | *form(A)* |
|  |  |  |  | Type VI secretion system proteins/ effectors | *vgrG, tssM, tssA, hcp1* |  |  |
|  |  |  | Adherence | Type I fimbriae | *fimB* |  |  |
|  |  |  |  | Flagella (cluster I) | *fliC* |  |  |
| MTR_ES05 | 16 | 10 | Secretion system protein | Type VI secretion system proteins/ effectors | *rhs, vgrG* | Macrolides | *mdf(A)* |
|  |  |  |  | Non-LEE encoded TTSS effectors | *espX4, espL1* | Aldehydes | *form(A)* |
|  |  |  |  | Type III secretion system proteins/ effectors | *spaP, spaQ* |  |  |
|  |  |  |  | Type VI secretion system proteins/ effectors | *vgrG* |  |  |
|  |  |  | Adherence | Type I fimbriae | *fimA, fimB, fimC, fimE, fimI* |  |  |
|  |  |  |  | Glucosephosphate uridylyltransferase | *galF* |  |  |
|  |  |  |  | Curli fibers | *csgA, csgB, csgC, csgD, csgE, csgF, csgG* |  |  |
|  |  |  | Immune evasion | ADP-L-glycero-D-mannoheptose-6-epimerase | *rfaD* |  |  |
| MTR_ET01 | 15 | 4 | Adherence | Type I fimbriae | *fimA, fimC, fimD, fimD, fimE, fimG, fimH* | Macrolides | *mdf(A)* |
|  |  |  |  | Curli fibers | *csgA, csgB, csgC, csgD, csgE, csgF, csgG* | Aldehydes | *form(A)* |
|  |  |  | Secretion system protein | Non-LEE encoded TTSS effectors | *espX4, espL1* |  |  |
|  |  |  |  | Type III secretion system proteins/ effectors | *spaP, spaQ, spaS* |  |  |
|  |  |  |  | Type VI secretion system proteins/ effectors | *rhs, vgrG* |  |  |
| MTR_ET06 | 16 | 6 | Adherence | *E. coli* common pilus (ECP) | *ecpR* | Macrolides | *mdf(A)* |
|  |  |  |  | Adhesin | *fdeC* | Aldehydes | *form(A)* |
|  |  |  |  | Type I fimbriae | *fimB, fimE* |  |  |
|  |  |  |  | Curli fibers | *csgA, csgB, csgC, csgD, csgE, csgF, csgG* |  |  |
|  |  |  | Secretion system protein | Type III secretion system proteins/ effectors | *spaP, spaQ* |  |  |
|  |  |  |  | Non-LEE encoded TTSS effectors | *espX4, espL1* |  |  |
|  |  |  |  | Type VI secretion system proteins/ effectors | *rhs* |  |  |
| MTR_ET08 | 16 | 6 | Adherence | *E. coli* common pilus (ECP) | *ecpR* | Macrolides | *mdf(A)* |
|  |  |  |  | Adhesin | *fdeC* | Aldehydes | *form(A)* |
|  |  |  |  | Type I fimbriae | *fimB, fimE* |  |  |
|  |  |  |  | Curli fibers | *csgA, csgB, csgC, csgD, csgE, csgF, csgG* |  |  |
|  |  |  | Secretion system protein | Non-LEE encoded TTSS effectors | *espX4, espL4* |  |  |
|  |  |  |  | Type III secretion system proteins/ effectors | *spaP, spaQ* |  |  |
|  |  |  |  | Type VI secretion system proteins/ effectors | *rhs* |  |  |
| MTR_ETO9 | 16 | 11 | Adherence | Type I fimbriae | *fimA, fimB, fimC, fimE, fimI* | Macrolides | *mdf(A)* |
|  |  |  |  | Curli fibers | *csgB, csgD, csgE, csgF, csgG* | Aldehydes | *form(A)* |
|  |  |  |  | Glucosephosphate uridylyltransferase | *galF ,galU* |  |  |
|  |  |  | Secretion system protein | Non-LEE encoded TTSS effectors | *espX4, espL1* |  |  |
|  |  |  |  | Type III secretion system proteins/ effectors | *spaP, spaQ* |  |  |
|  |  |  |  | Type VI secretion system proteins/ effectors | *rhs, vgrG* |  |  |
| MTR_ET11 | 17 | 5 | Adherence | *E. coli* common pilus (ECP) | *ecpR* | Macrolides | *mdf(A)* |
|  |  |  |  | Adhesin | *fdeC* | Aldehydes | *form(A)* |
|  |  |  |  | Curli fibers | *csgA, csgB, csgC, csgD, csgE, csgF, csgG* |  |  |
|  |  |  |  | Type I fimbriae | *fimB, fimE* |  |  |
|  |  |  | Secretion system protein | Non-LEE encoded TTSS effectors | *espX4, espL1, espL4* |  |  |
|  |  |  |  | Type III secretion system proteins/ effectors | *spaP, spaQ* |  |  |
|  |  |  |  | Type VI secretion system proteins/ effectors | *rhs* |  |  |
| MTR_E12 | 14 | 4 | Adherence | Type I fimbriae | *fimB, fimD, fimH* | Macrolides | *mdf(A)* |
|  |  |  |  | Curli fibers | *csgB, csgD, csgE, csgF, csgG* | Aldehydes | *form(A)* |
|  |  |  |  | Glucosephosphate uridylyltransferase | *galU* |  |  |
|  |  |  | Secretion system protein | Non-LEE encoded TTSS effectors | *espX4, espL1* |  |  |
|  |  |  |  | Type III secretion system proteins/ effectors | *spaP, spaQ* |  |  |
|  |  |  | Immune evasion | Outer membrane protein | *wza* |  |  |
|  |  |  |  | GDP-mannose dehydratase | *gmd* |  |  |
|  |  |  |  | GDP-fucose synthetase | *wcaG* |  |  |
