# Supplementary material for: Genome-wide analysis of extended-spectrum beta-lactamase-producing Escherichia coli from seafood in Bangladesh: population structure, resistome, virulome, and global dissemination patterns
Source: Front Microbiol. 2026 Feb 6;17:1737712. doi: 10.3389/fmicb.2026.1737712 (PMC12920492; doi:10.3389/fmicb.2026.1737712)
Supplement: SUPPLEMENTARY TABLE S7 — Summary of mobile genetic elements and plasmid content in ten E. coli strains. The table reports detected mobile elements, plasmid types, and the presence of associated antimicrobial resistance genes (ARGs) and virulence genes (VGs). [file Table_7.docx]

**Supplementary Table 7:** Summary of mobile genetic elements and plasmid content in ten Escherichia coli strains. The table reports detected mobile elements, plasmid types, and the presence of associated antimicrobial resistance genes (ARGs) and virulence genes (VGs).

| **Strains** | **Mobile Elements** | **ARGs** | **VGs** | **Plasmid** |
| --- | --- | --- | --- | --- |
| MTR_ECO2 | IS*Ec1*, IS*Ec17*, IS*3*, IS*Ec26*, IS*Ec5*, Tn*pR*_IS*Sba14*, IS*186B*, IS*609*, IS*1R*, IS*26*, **IS6100**, Tn*pA*_Tn*3*, IS*1A*, IS*Ec8*, IS*Kpn26*, IS*Lad1*, IS*911*, IS*30D*, IS*Ehe3*, IS*Ec48*, IS*Ec31*, IS*150*, IS*1F*, IS*1X2*, IS*621*, IS*421*, IS*2*, IS*682*, IS*Ec38*, IS*Ersp1*, MIT*EEc1*, IS*Kpn8*, IS*30* | ***qnrB4, dfrA17, qacE, mph(A), sul1, blaDHA-1****, sitABCD*, | *sitA, AslA, shiA, gad, nlpI, terC, fdeC, yehA, yehB, yehC, yehD, csgA, hlyE, ompT, fimH* | **IncFII, IncFIA, IncFIB(pHCM2), ColRNAI** |
| MTR_ECO3 | IS*Ec1*, IS*Kpn19*, IS*609*, IS*Kpn8*, IS*Ec5*, IS*Ec17*, IS*30*, IS*Kpn26*, MITEEc1, IS*102*, IS*903*, IS*26*, cn_6627_IS*102*, cn_6627_IS*903*, IS*1X2*, IS*1R*, IS*1H*, IS*903B*, IS*1A*, IS*150*, IS*Sfl10*, IS*30D*, IS*Ehe3*, IS*2*, IS*Ec27*, IS*Lad1*, IS*Ec26*, Tn*pA*_Tn*As1*, Tn*pR*_IS*Mex22*, IS*As17*, IS*Ec36*, Tn*pA*_Tn*3*, IS*Ecp1*, IS*Cro3*, IS*200C*, IS*Ersp1* | *tet(A), qnrS1, blaCTX-M-15* | *AslA, terC, yehA, yehB, yehC, yehD, gad, hlyE, nlpl, hha, fimH, csgA* | **Col440**I |
| MTR_ECO5 | IS*Ec1*, IS*Vsa17*, Tn*pR*_IS*Sba14*, IS*1G*, IS*186B*, IS*421*, IS*Ec5*, IS*1R*, IS*1A*, IS*Ec17*, IS*3*, IS*609*, IS*Cfr4*, IS*1S*, IS*Pre1*, IS*Eic2*, IS*Ehe3*, MITEEc1, cn_3874_IS*Ec17*, cn_4793_IS*Ec17*, cn_5418_IS*Ec17* | - | *clpK1, yehA, yehB, yehC, yehD, gad, nlpl, AslA, fimH, fdeC, csgA,* | **Col440I** |
| MTR_ESO5 | IS*Ec1*, IS*Kpn26*, IS*1R*, IS*1A*, IS*Ec17*, IS*3*, IS*150*, IS*1414*, IS*Ec52*, IS*Ec48*, IS*Eae2*, IS*2*, IS*1203*, IS*186B*, IS*421*, IS*Cro3*, IS*Ec26*, IS*Ec5*, IS*Ersp1*, IS*Kpn8*, MITEEc1, cn_5211_IS*Kpn26*, cn_4032_IS*3*, cn_26262_IS*3*, cn_48282_IS*Kpn26*, cn_1670_IS*Ec1* | - | *gad, hlyE, csgA, tia, terC, tibC, astA, tibA, AsIA, nlpI, ompT, fimH, yehA, yehB, yehC, yehD, hha* | - |
| MTR_ETO1 | IS*Ec1*, IS*Ec26*, IS*1R*, TnpR_IS*Pa38*, IS*Ec17*, IS*3*, IS*Ersp1*, IS*621*, IS*609*, Tn*pR*_IS*Sba14*, IS*Ec31*, IS*Cfr6*, IS*1H*, IS*Ec5*, MITEEc1, cn_8516_IS*621*, cn_29703_IS*621*, cn_7352_IS*3*, cn_11869_IS*3*, cn_1462_IS*3* | - | *lpfA, gad, hlyE, csgA, nlpl, yehA, yehB, yehC, yehD, fimH, terC, fdeC* | - |
| MTR_ETO6 | IS*Ec17*, IS*3*, IS*621*, IS*1H*, IS*609*, IS*Ec26*, IS*Ec1*, IS*Ersp1*, Tn*pR*_IS*Sba14*, IS*Ec5*, IS*Cfr6*, IS*Ec31*, MITEEc1, cn_29718_IS*621*, cn_36758_IS*621*, cn_44024_IS*621*, cn_8341_IS*Ec1* | - | *fimH, ompT, terC, yehA, yehB, yehC, yehD, gad, hlyE, nlpl, csgA, fdeC* | - |
| MTR_ETO8 | IS*Ec17*, IS*3*, IS*621*, IS*1H*, IS*609*, IS*Ec26*, IS*Ec1*, IS*Ersp1*, Tn*pR*_IS*Sba14*, IS*Ec5*, IS*Cfr6*, IS*Ec31*, MITEEc1, cn_29718_IS*621*, cn_36758_IS*621*, cn_44024_IS*621*, cn_8341_IS*Ec1* | - | *lpfA, gad, hlyE, csgA, nlpl, yehA, yehB, yehC, yehD, fimH, terC, fdeC, ompT* | - |
| MTR_ETO9 | IS*1203*, IS*2*, IS*Eae2*, IS*Ec48*, IS*Kpn26*, IS*Ec52*, IS1414, IS*186B*, IS*421*, IS*Ec17*, IS*3*, IS*Ec5*, IS*Ec26*, IS*609*, IS*Cro3*, IS*Ersp1*, IS*150*, IS*1A*, IS*1R*, IS*Kpn8*, MITEEc1, IS*Ec1*, cn_4032_IS*3*, cn_26262_IS*3*, cn_2244_IS*Ec1*, cn_1670_IS*Ec1*, cn_5211_IS*Kpn26*, cn_48282_IS*Kpn26* | - | *yehA, yehB, yehC, yehD, fimH, terC, hlyE, ompT, gad, terC, tia, fdeC, AslA, nlpI, tibC, tibA, astA, hha* | - |
| MTR_ET11 | IS*Ec17*, IS*3*, IS*621*, IS*1H*, IS*609*, IS*Ec26*, IS*Ec1*, IS*Ersp1*, Tn*pR*_IS*Sba14*, IS*Ec5*, IS*Cfr6*, IS*Ec31*, MITEEc1, cn_29718_IS*621*, cn_36758_IS*621*, cn_44023_IS*621*, cn_8341_IS*Ec1* | - | *csgA, lpfA, ompT, gad, yehA, yehB, yehC, yehD, nlpI, fimH, terC, fdeC, hlyE* | - |
| MTR_ET12 | IS*621*, IS*609*, IS*Ec1*, IS*Ec17*, IS*3*, IS*Ersp1*, IS*Ec26*, IS*1H*, MITEEc1, cn_43509_IS*621*, cn_27887_IS*621*, cn_18817_IS*621*, cn_29325_IS*621* | - | *csgA, nlpI, terC, gad, yehA, yehB, yehC, yehD, iss, fdeC, hlyE, lpfA, fimH* | - |

Legend: Bold elements are found in only the plasmids, and others in the chromosome carrying mobile elements
