# Supplementary material for: Genome-wide analysis of extended-spectrum beta-lactamase-producing Escherichia coli from seafood in Bangladesh: population structure, resistome, virulome, and global dissemination patterns
Source: Front Microbiol. 2026 Feb 6;17:1737712. doi: 10.3389/fmicb.2026.1737712 (PMC12920492; doi:10.3389/fmicb.2026.1737712)
Supplement: Supplementary file 13 [file Table_9.docx]

**From:**[biosamplehelp@ncbi.nlm.nih.gov](mailto:biosamplehelp@ncbi.nlm.nih.gov) <[biosamplehelp@ncbi.nlm.nih.gov](mailto:biosamplehelp@ncbi.nlm.nih.gov)>
**Date:**Sunday, 5 October 2025 at 16:30
**To:**Refath Farzana <[refath.farzana@biology.ox.ac.uk](mailto:refath.farzana@biology.ox.ac.uk)>
**Subject:**BioSample accessions SAMN52392760, SAMN52392761, SAMN52392762, SAMN52392763, SAMN52392764, SAMN52392765, SAMN52392766, SAMN52392767, SAMN52392768, SAMN52392769

Dear Refath Farzana,

This is an automatic acknowledgment that your recent submission to the BioSample database has been successfully processed and will be released on the date specified.

BioSample accessions:                   SAMN52392760, SAMN52392761, SAMN52392762, SAMN52392763, SAMN52392764, SAMN52392765, SAMN52392766, SAMN52392767, SAMN52392768, SAMN52392769
Temporary SubmissionID: SUB15686303
Release date:                   2027-11-30, or with the release of linked data, whichever is first

A submission summary and the links by which your BioSample records will be accessible are appended and attached.


Please reference BioSample accessions SAMN52392760, SAMN52392761, SAMN52392762, SAMN52392763, SAMN52392764, SAMN52392765, SAMN52392766, SAMN52392767, SAMN52392768, SAMN52392769 when making corresponding sequence data submissions.

Send questions and update requests to [biosamplehelp@ncbi.nlm.nih.gov](mailto:biosamplehelp@ncbi.nlm.nih.gov); include the BioSample accessions SAMN52392760, SAMN52392761, SAMN52392762, SAMN52392763, SAMN52392764, SAMN52392765, SAMN52392766, SAMN52392767, SAMN52392768, SAMN52392769 in any correspondence.

Regards,

NCBI BioSample Submissions Staff
Bethesda, Maryland USA
***********************************************************
(301) 496-2475
(301) 480-2918 (Fax)
[biosamplehelp@ncbi.nlm.nih.gov](mailto:biosamplehelp@ncbi.nlm.nih.gov) (for BioSample questions/replies)
[info@ncbi.nlm.nih.gov](mailto:info@ncbi.nlm.nih.gov) (for general questions regarding NCBI)
***********************************************************

Accession    Sample Name    SPUID    Organism    Tax ID    Strain   
SAMN52392760    T9.fasta    T9.fasta    Escherichia coli    562    T9.fasta   
SAMN52392761    C2.fasta    C2.fasta    Escherichia coli    562    C2.fasta   
SAMN52392762    C3.fasta    C3.fasta    Escherichia coli    562    C3.fasta   
SAMN52392763    C5.fasta    C5.fasta    Escherichia coli    562    C5.fasta   
SAMN52392764    R5.fasta    R5.fasta    Escherichia coli    562    R5.fasta   
SAMN52392765    T1.fasta    T1.fasta    Escherichia coli    562    T1.fasta   
SAMN52392766    T6.fasta    T6.fasta    Escherichia coli    562    T6.fasta   
SAMN52392767    T8.fasta    T8.fasta    Escherichia coli    562    T8.fasta   
SAMN52392768    T11.fasta    T11.fasta    Escherichia coli    562    T11.fasta   
SAMN52392769    T12.fasta    T12.fasta    Escherichia coli    562    T12.fasta   


<https://www.ncbi.nlm.nih.gov/biosample/52392760>
<https://www.ncbi.nlm.nih.gov/biosample/52392761>
<https://www.ncbi.nlm.nih.gov/biosample/52392762>
<https://www.ncbi.nlm.nih.gov/biosample/52392763>
<https://www.ncbi.nlm.nih.gov/biosample/52392764>
<https://www.ncbi.nlm.nih.gov/biosample/52392765>
<https://www.ncbi.nlm.nih.gov/biosample/52392766>
<https://www.ncbi.nlm.nih.gov/biosample/52392767>
<https://www.ncbi.nlm.nih.gov/biosample/52392768>
<https://www.ncbi.nlm.nih.gov/biosample/52392769>
